# WebCSEA: web-based cell-type-specific enrichment analysis of genes

**DOI:** 10.1093/nar/gkac392

**Published:** 2022-05-24

**Authors:** Yulin Dai, Ruifeng Hu, Andi Liu, Kyung Serk Cho, Astrid Marilyn Manuel, Xiaoyang Li, Xianjun Dong, Peilin Jia, Zhongming Zhao

**Affiliations:** Center for Precision Health, School of Biomedical Informatics, The University of Texas Health Science Center at Houston, Houston, TX 77030, USA; Center for Precision Health, School of Biomedical Informatics, The University of Texas Health Science Center at Houston, Houston, TX 77030, USA; Center for Advanced Parkinson Research, Brigham and Women's Hospital, Harvard Medical School, Boston, MA 02115, USA; Genomics and Bioinformatics Hub, Department of Neurology, Brigham and Women's Hospital, Harvard Medical School, Boston, MA 02115, USA; Center for Precision Health, School of Biomedical Informatics, The University of Texas Health Science Center at Houston, Houston, TX 77030, USA; Department of Epidemiology, Human Genetics and Environmental Sciences, School of Public Health, The University of Texas Health Science Center at Houston, Houston, TX 77030, USA; Center for Precision Health, School of Biomedical Informatics, The University of Texas Health Science Center at Houston, Houston, TX 77030, USA; Center for Precision Health, School of Biomedical Informatics, The University of Texas Health Science Center at Houston, Houston, TX 77030, USA; Center for Precision Health, School of Biomedical Informatics, The University of Texas Health Science Center at Houston, Houston, TX 77030, USA; Department of Biostatistics and Data Science, School of Public Health, The University of Texas Health Science Center at Houston, Houston, TX 77030, USA; Center for Advanced Parkinson Research, Brigham and Women's Hospital, Harvard Medical School, Boston, MA 02115, USA; Genomics and Bioinformatics Hub, Department of Neurology, Brigham and Women's Hospital, Harvard Medical School, Boston, MA 02115, USA; Center for Precision Health, School of Biomedical Informatics, The University of Texas Health Science Center at Houston, Houston, TX 77030, USA; Center for Precision Health, School of Biomedical Informatics, The University of Texas Health Science Center at Houston, Houston, TX 77030, USA; MD Anderson Cancer Center UTHealth Graduate School of Biomedical Sciences, Houston, TX 77030, USA

## Abstract

Human complex traits and common diseases show tissue- and cell-type- specificity. Recently, single-cell RNA sequencing (scRNA-seq) technology has successfully depicted cellular heterogeneity in human tissue, providing an unprecedented opportunity to understand the context-specific expression of complex trait-associated genes in human tissue-cell types (TCs). Here, we present the first web-based application to quickly assess the cell-type-specificity of genes, named Web-based Cell-type Specific Enrichment Analysis of Genes (WebCSEA, available at https://bioinfo.uth.edu/webcsea/). Specifically, we curated a total of 111 scRNA-seq panels of human tissues and 1,355 TCs from 61 different general tissues across 11 human organ systems. We adapted our previous decoding tissue-specificity (*deTS*) algorithm to measure the enrichment for each tissue-cell type (TC). To overcome the potential bias from the number of signature genes between different TCs, we further developed a permutation-based method that accurately estimates the TC-specificity of a given inquiry gene list. WebCSEA also provides an interactive heatmap that displays the cell-type specificity across 1355 human TCs, and other interactive and static visualizations of cell-type specificity by human organ system, developmental stage, and top-ranked tissues and cell types. In short, WebCSEA is a one-click application that provides a comprehensive exploration of the TC-specificity of genes among human major TC map.

## Graphical Abstract

**Graphical Abstract:**
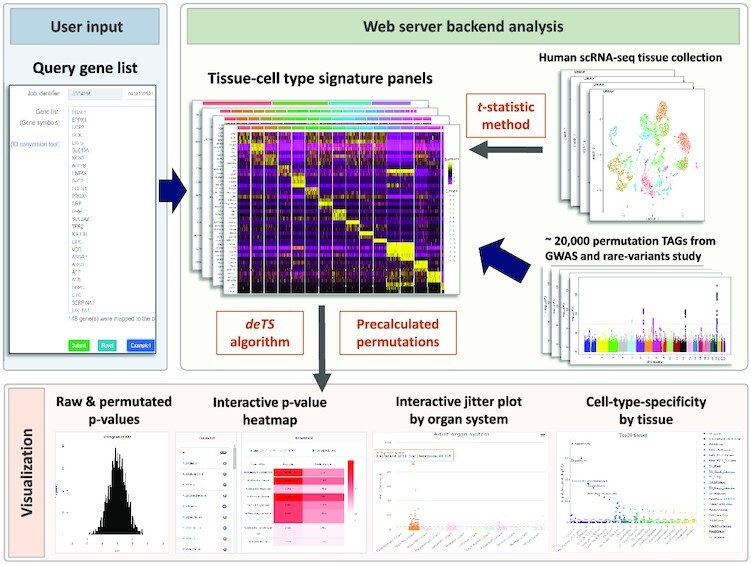
WebCSEA takes a simple gene list as the input. After the backend cell-type-specific-enrichment analysis, the enrichment results will be visualized in interactive heatmap and jitter plots by human organ system and tissue in minutes.

## INTRODUCTION

When we prioritize a list of risk genes from complex trait and common disease studies, we always consider the context in which these genes might manifest their impact on human tissue-cell types. With the rapid development of single-cell technology, millions of cellular transcriptomes have been generated by multiple international consortia [e.g. Human Cell Atlas (Fetal) (1), Tabula Sapiens (2) and Human Cell Landscape (3–5)] to characterize the cellular heterogeneity in human major tissues and organ systems among different developmental stages. To harness these large-scale heterogeneous data, there is a pressing need for advanced computational methods and web services that could characterize the signature genes of human cell types in different contexts and, reversely, assess the cell types for which a particular gene set may be enriched. To the best of our knowledge, there are a few databases that provide comprehensive cell marker curation, including PanglaoDB (6), CellMarker (7), CellKb (8) and GeneMarkeR (9). However, these methods and tools were designed mainly for curating the marker genes for each cell type, and some of these databases either require a commercial license or have been out of date for several years. Among them, only PanglaoDB provides a reverse search for the cell type enriched by input genes with limited characters. The Specific Expression Analysis (SEA) tool only provided enrichment analysis by using RNA-seq or microarray data from mouse and human central nervous system (http://genetics.wustl.edu/jdlab/csea-tool-2/). Currently, no online web server systematically provides comprehensive human TCs by leveraging scRNA-seq data. Thus, novel methods and tools are in demand to explore tissue-cell-type- (TC-) and context-specificity of gene lists derived from diverse scenarios, e.g. genetic studies of complex disease, differentially expressed genes from bulk high-throughput profiling, previous tissue-specific curation, functional pathway/terms, among others. To fill this gap, we have published a series of works in decoding tissue- and cell-type- specificity of genes, including algorithm development (10,11) as well as databases curation for the large-scale well-annotated transcriptome data at both tissues and cell type level (12–15).

**Figure 1.**
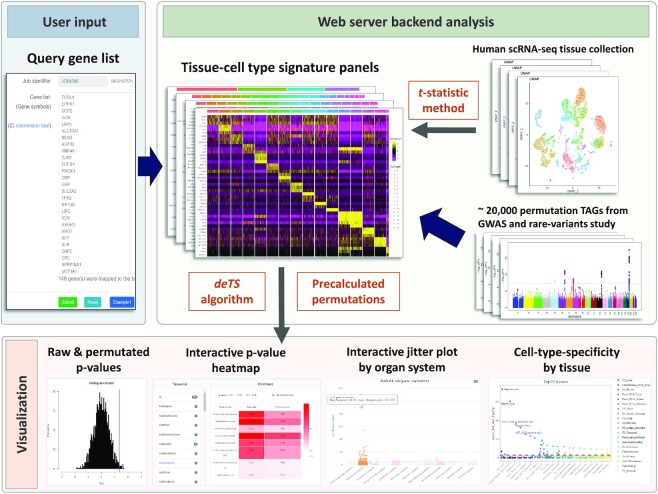
Framework of WebCSEA. WebCSEA web server has three major parts: user input (blue background), web server backend analysis (green background), and result visualization (red background). 1) WebCSEA takes a simple gene list as the input. 2) The web server backend analysis includes cell-type-specific enrichment analysis (CSEA, *deTS* algorithm) for query gene list and tissue-cell type signature panels curated from >100 of human single-cell RNA-sequencing data using the *t*-statistic method. Then, a permutation test will be conducted to adjust the raw *P-*value with the precalculated permutations of ∼20 000 trait-associated gene sets (TAGs) using CSEA for each tissue-cell type. 3) Once CSEA and subsequent permutation test in the backend is complete, WebCSEA will provide visualization for both raw and permutated *P-*values by using interactive *P-*value heatmap and jitter plots by human organ system and tissue in a few minutes.

In this work, we aim to provide a user-friendly interactive platform for a broad set of users or investigators to explore the cellular context of any given gene list, like the gene-set enrichment analysis for Gene Ontology and the KEGG pathway (Figure 1). The accurate assessment of context-specificity requires us to tackle the following challenges: (i) comprehensive collection of human tissue-cell type panels that covers the major human organ systems, tissues, cell types and different developmental stages; (ii) we need to make our cell-type specificity enrichment analysis (CSEA) results comparable among different tissue cell types, considering potential biases due to different lengths of signature genes among tissue-cell types; (iii) we need to consider the extent of reliability for our WebCSEA results. We benchmarked our tool with a couple of known tissue- and cell-type-specific signature genes to evaluate the performance.

## MATERIALS AND METHODS

### Human scRNA-seq tissue panel collection and ontology annotation

**Figure 2.**
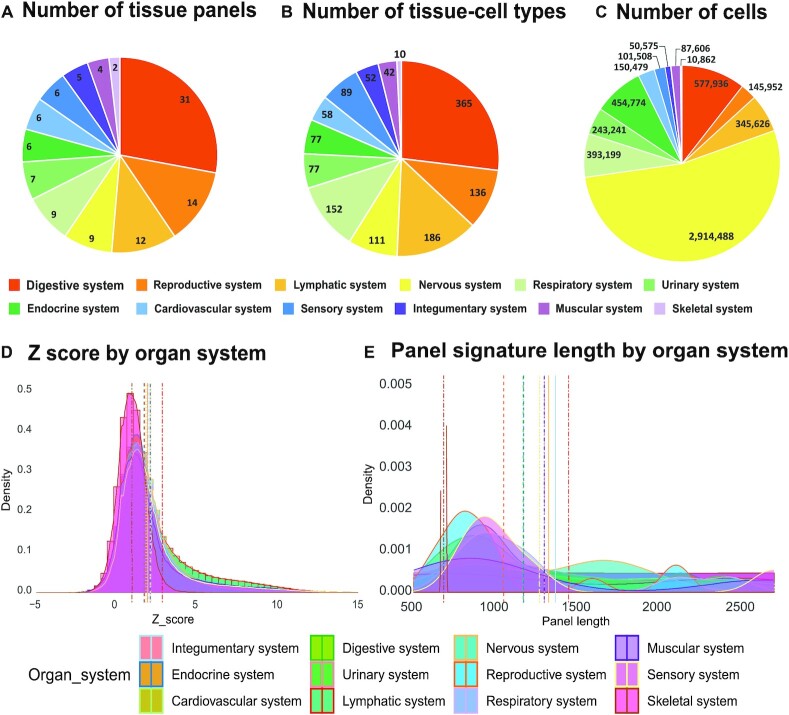
Summary for data curation for constructing our tissue-cell type signature panel and permutation test. In the pie charts A, B and C, each color represents one human organ system; (**A**) shows the number of tissues collected in each organ system. (**B**) shows the number of tissue-cell types collected in each organ system. (**C**) shows the total number of cells collected in each organ system. In the density plot of D&E, each color represents one human organ system, and the vertical line in the corresponding color represents the mean of Z score of each organ system. (**D**) For each organ, we plot the density distribution and mean of the permutation *z* scores pre-calculated by *deTS* algorithm using the ∼20 000 genetic trait-associated gene sets (TAGs) and single-cell signature panels. (**E**) For each organ, we plot the density distribution and mean of panel signature length by organ system.

Human bodies are composed of 11 major organ systems, ∼100 organs/tissues and >100 unique cell types or thousands of sub-cell types (16). To unveil the cellular heterogeneity of these diverse human tissues, we harnessed human organ transcriptome profiling recently generated by leading consortiums such as Human Cell Atlas (1), Human Cell Landscape (5), and Tabula Sapiens (2,12), and a few representative studies (3,4,17–22). Overall, we collected >5.5 million cells from 111 tissue panels and 1355 tissue-cell types (TCs), which belong to 61 general tissues from different developmental stages across all 11 human organ systems (16) (Figure 2A–C). Given the diverse cell types between the central nervous system and sensory nervous system, we split them into two different systems for visualization. Hence, we have grouped the 111 tissue panels into 12 organ systems in our WebCSEA platform (Figure 2A). Since we adapted original cell type annotations from each panel in our enrichment analysis, the same tissue-cell type (TC) might be potentially annotated to different cell type names in different studies. Therefore, we created general traceable ontology IDs for each tissue and cell type to make TCs from different studies comparable. We adapted our previous strategy used in CSEA-DB to annotate the tissues using the Uberon anatomy ontology (23) and cell type Ontology ID with Cell Ontology (https://www.ebi.ac.uk/ols/ontologies/cl, accessed 2/15/2022) (24,25). Details of the process are described in the Methods section of our previous work (13).

We filtered out lowly expressed genes and used our in-house *t*-statistic-based method to identify the tissue-cell-type signature genes (11). Notably, there are 25 tissues with at least two panels from different developmental stages or studies, including those tissues targeted by disease with high prevalence, such as lung (6 panels), pancreas (6 panels), liver (5 panels), heart (4 panels) and eye (4 panels). Due to the sampling issue, single-cell RNA-seq data may miss some cell types of the tissue. These ‘redundant’ tissues in our curation provide an opportunity for both validating the findings and comprehensive observation.

### Application of cell type-specific enrichment analysis

We modified our previously developed ‘tissue-specific enrichment analysis’ and applied it to explore the cell type-specificity within each tissue. Details of methods could be found in the Method part of our previous work (11,13). Briefly, we used the log_2_ (CPM + 1) normalized single-cell transcriptome matrix to calculate the cell type-specific expression within the cell types (number of cells ≥ 30) in each tissue. Then, we built a linear regression model to measure the TC-specificity of *i* gene in *j* cell type by using the coefficient (*t*-statistics) of each gene. We defined the top 5% *t*-statistic score genes in focal cell type as the cell-type-specific genes. Lastly, Fisher's exact test was conducted to assess whether the trait-associated gene (TAG) set from each trait is overrepresented with the cell type-specific genes, where the *P-*value indicates the significance of this cell-type specificity enrichment analysis (CSEA).

### Permutation-based cell-type specificity test using genetic TAGs

To address the common biased problem raised by different lengths of signature genes and input gene lists during the enrichment analysis, we developed a permutation-based method to adjust the *P-*value from overrepresentation analysis. One straightforward approach will be sampling the random gene sets purely from all genes expressed in each TC and calculating CSEA for them thousands and millions of times to obtain the permutated *P-*values. However, this method will require a lot of computational resources and surfer from the inflation of Type I error rate since the null hypothesis that TC-specific enrichment is no better than random sampling results is relatively loose and easy to reject and, therefore, inappropriate. Instead, we decided to define a more biologically meaningful null hypothesis for assessing the tissue and cell-type specificity, given the nature that trait-associated gene sets (TAGs) from human complex traits and diseases have ‘moderate’ tissue- and cell-type- specificity among different human tissue-cell types (26). Intuitively, if one gene list has a CSEA raw *P-*value ranked as among the top of the *P-*values from CSEA for large-scale random TAGs, then we reject the null hypothesis that this gene list has no enrichment with this tissue cell type.

Our curation for TAGs has two major components, European Ancestry genome-wide association studies (GWAS) from our long-term curation of GWAS catalog described in TSEA-DB and CSEA-DB (13,15), and rare-variants gene-based association phenotypes from UK biobank (27).

For GWAS TAGs, we used Multi-marker Analysis of GenoMic Annotation (MAGMA v1.07) (28) to calculate the gene-level *P-*values. Briefly, we used all SNPs within the 50 kb upstream and 35 kb downstream window of each gene region and then calculated the mean χ^2^ statistic for these SNPs to obtain gene-based *P-*values. Lastly, we accounted for the effects of the gene length, SNP density, and local linkage disequilibrium structure (1000 Genome Project Phase 3 European population reference panel). We used a dynamic gene-based *P-*value threshold to generate the TAGs for >5600 GWAS from our previous curation and GWAS catalog accessed by 25 November 2021 (13,29). Consistent with the input gene length, we further limited the number of genes in TAGs from 20 to 2000. In the end, we collected 17 807 GWAS TAGs.

For the TAGs from rare-variants gene-based associations, we obtained Hail matrix table format (https://github.com/hail-is/hail/releases/tag/0.2.13) results from Genebass (gene-biobank association summary statistics) database, which contains associations for over 3700 phenotypes from 281 850 individuals in the UK Biobank (27). Considering different scenarios of causal variant effects, they conducted the kernel association test (SKAT test) (30), SKAT optimal unified test (SKAT-O test) (31), and burden test (30) to assess three types of rare variants (loss of function (LoF), missense and synonymous) in each gene. We used the normal significant 1 × 10^–5^*P*-value thresholds for the burden test, SKAT test, and SKAT-O test. After filtering the gene length ranges from 20 to 2000, we collected 1856 TAGs from rare-variants gene-based associations.

We conducted >26 million times of CSEA for 19 663 TAGs over 1355 TCs as precalculated permutations for backend analysis (Figure 1). To assess the distribution of these permutated *P-*values, we converted these *P*-values to Z score by the cumulative distribution function. As shown in Figures 2D and E, the largest and smallest *Z* scores in the organ systems are the lymphatic system (2.81) and skeletal system (0.93), respectively. Similarly, the longest and shortest mean panel lengths are the lymphatic system (1513.45) and skeletal system (764.38), respectively. Thus, we observed a positive correlation between the mean *Z* score by organ system and the panel signature length by organ system, confirming the aforementioned bias from the signature length in each TC. Therefore, within each TC, the relative rank among these 19 663 TAG *P-*values was used to depict the TC-specificity.

We followed the formulas below for each inquiry gene list and conduct the CSEA across 1355 TCs to get the permutated *P-*values by ranking among the *P-*values in 19 663 TAGs in each TCs. Then, we used Fisher's method to combine an overall *P-*value from the permutated *P-*values by TAGs and TCs.

(1) $$\begin{eqnarray*}{{{P}}_{{\rm{perm}}}}\left( {\rm{i}} \right) &=& {\rm rank} \left( {{{{P}}_{{\rm{raw}}}}\left( {\rm{i}} \right){\rm{,}}{{{P}}_{{{\# TAG}}}}\left( {\rm{i}} \right)} \right){\rm{/ 19,663}}\nonumber\\ &&\times cumulative factor\end{eqnarray*}$$

(2) $$\begin{equation*}{{{P}}_{{\rm{TC}}}}\left( {\rm{i}} \right){\rm{ = rank }}\left( {{{{P}}_{{{raw}}}}\left( {\rm{i}} \right){\rm{,}}{{{P}}_{{{\# TC}}}}\left( {\rm{i}} \right)} \right){\rm{/ 1,355}}\end{equation*}$$

(3) $$\begin{equation*}X_4^2\sim - 2\,{\rm{I}}n\left( {{P_{perm}}\left( {\rm{i}} \right)*{P_{TC}}\left( {\rm{i}} \right)} \right)\end{equation*}$$

Specifically, *i* indicates the *i*th TC, where *P*_raw_(*i*) is the hypergeometric test of inquiry gene list and TC-specific genes by our previous method decoding tissue-specificity (*deTS*); the cumulative factor is an empirical parameter used to adjust the TAGs length difference. Here we used the proportion of TAGs in a total of 19 663 TAGs with at least the length of input gene list; *P*_#TAG_(*i*) represents the vector of permutated CSEA *P-*values for 19 663 TAGs; Then, the *P*_raw_(*i*) is further ranked overall the 1355 CSEA *P*_#TC_(*i*) in *i*th TC. Lastly, we used Fisher's method to combine *P-*values from *P*_perm_(*i*) and PTC(*i*) in ith TC into one chi-squared distribution statistic (*X2*). The sum of two independent chi-squared values follows a chi-squared distribution with 2*2 degrees of freedom.

### Web server specifications

#### Input

**Figure 3.**
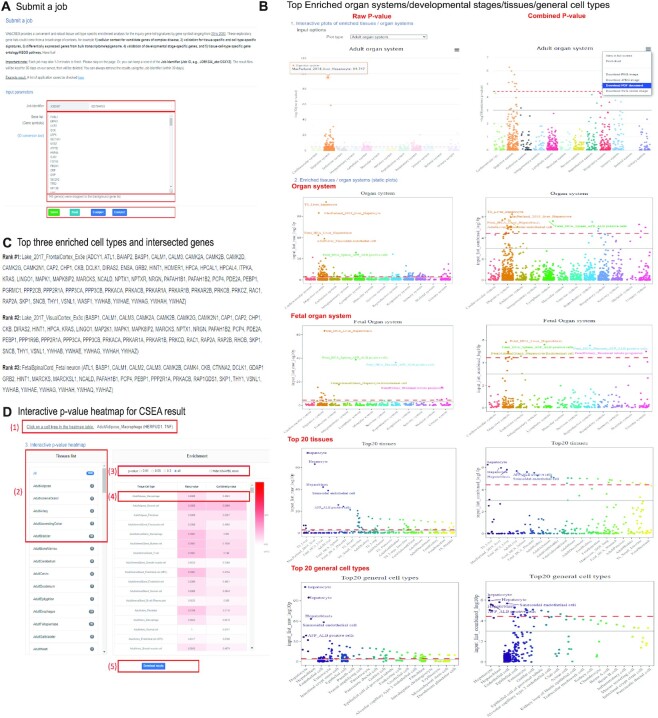
Detail tutorials for WebCSEA result visualization. (**A**) Input page, the red rectangles highlight four major functions in submitting a gene list, including job identifier, gene list, number of genes mapped to the background genes, and function buttons to submit the gene list, to reset the input, and to use the example gene list. (**B**) jitter plots for CSEA results of –log_10_ raw (left) and combined *P-*values (right) among 1355 tissue-cell types by five categories, including adult organ system, organ system, fetal organ system, top 20 tissues, and top 20 general cell types, respectively. The red dashed line indicates the Bonferroni-corrected significance (*P* = 3.69 × 10^–5^) by 1355 tissue-cell types. The grey solid line indicates the nominal significance (*P* = 1 × 10^–3^). 1) Interactive plots adult stage organ system; when the cursor is placed at each dot, the dots in that specific column will all be highlighted and the detailed TC information, as well as the –log10 (P) in that specific category, will be displayed in the floating window. The corresponding plot could be downloaded in JPEG, SVG, and PDF format. The users could further highlight the TCs of interest on those downloaded files. 2) Static jitter plots; each dot represents one tissue-cell type in the group on the x-axis differentiated by color. (**C**) Top three enriched cell types and their intersected genes with gene signatures of corresponding cell types. (**D**) The red rectangles highlight five functions of interactive heatmap. 1) This part will interactively show the intersected genes of input genes and selected the tissue-cell types; 2) Clicking the listed tissue panels will interactively show the corresponding tissue panel raw and combined *P-*values; 3) Filtration of *P-*values at different thresholds; 4) The color of the heatmap is proportional to the raw and combined *P-*values from CSEA results of inquiry gene list. Each row represents one tissue-cell type. 5) Download button allows the users to download all the CSEA results and visualizations.

WebCSEA only requires a gene symbol list as the input, which could take up to 3 min for accomplishing the job for 1000 genes (Figure 3A). A job identifier is provided for each job for the user to retrieve and share the results. After the user input the gene list, the system will automatically count the genes mapped to our background reference gene symbols curated from GRCh38 Ensembl version 99. We provide three basic function buttons to submit a job, reset input, and use an example gene list, respectively. To get a meaningful cell-type-specific analysis, we suggest the users input a gene list with a length ranging from 20 to 2000.

#### Output

We provide the user an instant overview of top TC-specificity results of a query gene list by jitter plots the enrichment results for both raw *P-*value and combined *P-*value among the 1355 TCs by five categories of stratification, including adult organ system, organ system, fetal organ system, top 20 tissues, and top 20 general cell types from up to bottom (Figure 3B) in both interactive and static plots. The interactive jitter plot function allows the user to explore the specific tissue-cell-type-specificity freely for both raw and combined *P-*values. Specifically, we provided several input options to display the cell-type-specificity enrichment results, including organ systems, adult and fetal developmental stages, top 20 tissues, and top 20 general cell types. When the cursor is placed at each dot, the dots in that specific column will all be highlighted and the detailed tissue cell type information, as well as the –log_10_(*P*) in that specific category, will be displayed in the floating window. The user could further download the corresponding plot in JPEG, SVG and PDF format. The users could further highlight the tissue-cell type of interest on those downloaded files. For the static jitter plots, we annotated the top 5 TCs in each category of stratification. We highlighted the different components from each category by color. We set two different thresholds to indicate the significance. The red dashed line is Bonferroni-corrected significance (*P* = 3.69 × 10^–5^) by 1355 tissue-cell types. The solid grey line is the nominal significance (*P* = 1 × 10^–3^). The top 3 enriched TCs in combined *P-*value were listed with their intersected genes between the input gene list and the corresponding cell type gene signatures (Figure 3C). Lastly, we used an interactive *P-*value heatmap to display the enrichment result in the overview model or tissue panel model (Figure 3D). The user could easily browse and filter the detailed *P-*values of the top-ranked tissue panels or tissue of their interest. More importantly, the user could click the heatmap to obtain the intersected genes of input genes and selected TCs. Lastly, we also provide the download link for the full results and the Github repository to reproduce or customize the visualization.

### Web service design

The web server runs on a Linux server equipped with CentOS 7 and Apache (version 2.4). The server has four CPUs [Intel(R) Xeon(R) E5-2637 v3] with 128GB memory and an 8TB hard disk to support computational tasks. Our WebCSEA follows the Model-View-Controller (MVC) web design framework. The model is the main calculation function on the backend implemented in the R programming language utilizing the *deTS* R package. The calculation function takes the input data submitted and generates the result files. The views are the frontend interfaces that were implemented using HTML5, CS and JavaScript. In WebCSEA, the view pages are designed to guide users to input their data properly, show job running status, display feedback including practical errors, and present results when the job finishes successfully. The controller leveraged Ajax technology to validate the inputs, submit jobs, check running status, retrieve result data from the server-side, and update the information in views without refreshing the web page by utilizing the jQuery library. PHP was also used as an auxiliary function for data display. The MVC design provides a clear separation of logic, making the testability frictionless and allowing us to extend functions and add new features easily in the future. We designed a queue strategy to organize the submitted jobs to avoid server overloading or task jams. Each user can submit as many jobs as necessary. WebCSEA allows a maximum of 5 jobs running in parallel, and other jobs will be automatically added to a queue.

### Benchmark examples and context of application

**Figure 4.**
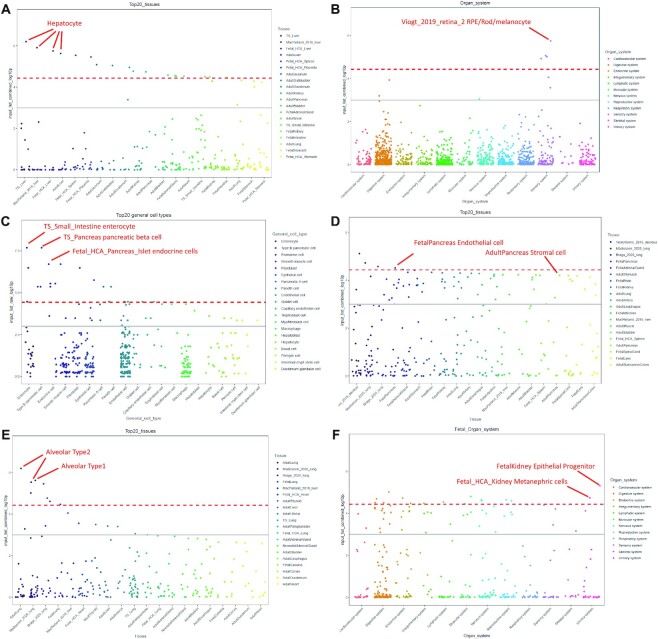
Visualization of *P-*values calculated by WebCSEA among 1355 tissue-cell types. The jitter plots from (**A**) to (**F**) are the specificity of queried genes among tissue-cell types (TCs) by different stratification strategies from WebCSEA. Each dot represents one tissue-cell type in the group on the x-axis differentiated by color. The most significant dots are highlighted and annotated with their corresponding TCs. The red dashed line indicates the Bonferroni-corrected significance (*P* = 3.69 × 10^–5^) by 1355 TCs. The grey solid line indicates the nominal significance (*P* = 1 × 10^–3^). The X-axis represents the components in different stratification strategies. Y-axis indicates the (–log_10_ (combined *P-*value)) or (–log_10_ (raw *P-*value)) for each TC from CSEA result.

We provided extensive examples and applications in the ‘Tutorial’ pages. There, we evaluated the performance of our WebCSEA by using a few ground truth gene lists from cell-type signature genes, disease risk genes with unknown onset tissues and cell-type-specific pathways. As shown in Figure 4A, we obtained the 110 hepatocyte-specific expressed genes from the cell-type signature genes database PanglaoDB (6). We could observe the top four dots were all annotated as hepatocytes from four different scRNA-seq panels, suggesting the high accuracy and consistency of our WebCSEA. Moreover, this also indicated our WebCSEA could help to validate cell type signature genes. Figure 4B shows 119 eye-diseases related genes from the RetNet (https://sph.uth.edu/retnet/). We could see a peak in the Sensory system only, aligning with the disease context. In Figure 4C, we used the 22 gold standard type 2 diabetes GWAS risk genes from T2D Knowledge Portal (https://t2d.hugeamp.org/). The top 3 significant enriched TCs are small intestine enterocyte, pancreas pancreatic beta cell, and fetal pancreas islet endocrine cells, respectively. These findings indicate that WebCSEA could identify the disease-relevant tissue and cell types by using a limited number of gold standard genes. Next, we raised a few scenarios in which the users could use the WebCSEA to explore the TC-specificity of focal gene lists, including differentially expressed genes (DEG) from Bulk RNA-seq, tissue-marker genes, and developmental stage-specific genes. In Figure 4D, we conducted the CSEA for the 190 human homologues derived from the DEG analysis of pancreatic ductal adenocarcinoma mouse with CD73 inhibitor AB680 treatment between controls (32). We identified two major cell types (endothelial cell and stromal cell) enriched in the pancreas, which shed light on exploring the drug mechanism at the cellular level. As shown in Figure 4E, we adapted the 104 signature genes of TCGA lung adenocarcinoma (LUAD) from tissue-specific gene database in cancer database (TissGDB) (33). We successfully identified the tissue marker genes were mainly contributed by Alveolar Type 2 and Alveolar Type 1 cells in multiple tissue panels. Figure 4F shows the CSEA result of 471 Kidney developmental genes derived from the previous study (34). We identified epithelial progenitor and metanephric cells highlighted in the fetal stage, which could help validate the developmental stage of this gene list. In addition, WebCSEA accurately explores TC-specific gene ontology pathways. We identified neurons that were enriched with Excitatory neuron-specific pathway (Figures 3B, C) curated from the previous studies (35,36).

## CONCLUSION

WebCSEA (Web-based Cell-type Specific Enrichment Analysis of Genes) provides a gene set enrichment query among a systematic collection of human tissue-cell type expression signatures with manual ontology curation. We leverage our *deTS* algorithm over 1355 human tissue-cell types and generate an enrichment *P-*value to assess their specificity for each query gene list. We further calculate a permutation-test *P-*value with ∼20 000 previously calculated *P-*values derived from TAGs of diverse human diseases and traits, which provides us a natural reference for the goodness of enrichment adjusted with the length difference in signature genes of different cell types. We provide extensive visualization functions such as interactive heatmap, and interactive and static jitter plots to display the cell-type specificity across 1355 human TCs by human organ system, developmental stage, and top-ranked tissues and cell types. We also provide intersected genes of the most enriched tissue-cell types as well as any tissue-cell types of interest by clicking the interactive heatmap. Furthermore, all the results and visualization in WebCSEA could be downloaded and reproduced. Users can filter, prioritize and customize the visualization of CSEA results with the download materials and script on Github. Lastly, we validated the WebCSEA performance using gold-standard gene lists and obtained high accuracy and consistency. In summary, WebCSEA platform provides a quick, accurate, and comprehensive assessment for exploring and validating the context- and TC-specificity of focal gene list freely. Moreover, WebCSEA results might shed light on identifying unknown pleiotropic effects of genes on some ‘unexpected’ TCs, which potentially help to explain the comorbidities of complex diseases that have shared genetic components and disease genes (37). Just like the gene-set enrichment analysis, we expect WebCSEA to serve the broad users in both research and clinical community to explore the biological context of human genes.

There are a few limitations that could be improved upon in the future. Although we covered 61 human tissues across fetal, neonatal, and adult stages, many tissues and contexts still have not been included. With the exponential growth of single-cell research, we expect a more comprehensive collection of tissues to be generated and available in our WebCSEA. We are aware that the raw *P-*value will become more significant along with the increasing length of the input gene list and the incidence of false-positive results. Hence, we utilized the weak tissue-specific TAG permutations to account for the ‘inflated’ *P-*values in TCs with long signature genes. However, this correction might be too stringent when the input gene list is small (∼20 genes). Thus, we suggest that users take both raw *P-*value and permutated *P-*value into consideration to depict the TC-specificity. Lastly, the significant enrichment result from WebCSEA could only be interpreted as an association among thousands of TCs. We expect these preliminary results could inspire and support the users to conduct follow-up studies to further uncover the cellular context of the given gene list.

## DATA AVAILABILITY

All the data generated or analyzed in this study are available from the authors, upon request. The enrichment method *deTS* has been deposited in CRAN repository and all the plots from the WebCSEA could be reproduced with the code on Github repertoire https://github.com/davidroad/WebCSEA.
